# Assessing cognitive impairment in home-dwelling Chinese elders aged 80+: a detailed survey of 13,000 participants focusing on demographic factors, social engagement, and disease prevalence

**DOI:** 10.3389/fpsyt.2024.1355708

**Published:** 2024-04-02

**Authors:** Sensen Bian, Xiaobing Tian, Fanli Meng, Chunjie Xu, Yan Zhao, Qian Gao, Chengzhi Bian

**Affiliations:** ^1^Department of Public Health, Hangzhou Normal University, Hangzhou, Zhejiang, China; ^2^Nanjing Zhiyuan Healthcare Training Center, Jiangsu SuYi Health Care Research Institute, Nanjing, Jiangsu, China; ^3^Health management center, The First Affiliated Hospital of Hainan Medical University, Haiko, Hainan, China

**Keywords:** socio-economic, social interaction, diabetes, cognition function, mini-cog

## Abstract

**Introduction:**

Cognitive Impairment (CI) in the elderly, encompassing conditions ranging from Mild Cognitive Impairment (MCI) to dementia, represents a growing public health concern globally. This study aims to investigate the prevalence and correlates of CI among individuals aged 80 and above.

**Methods:**

The study conducts 13,027 elderly individual’s door-to-door surveys, followed by the cross-tabulation of analysis data, logistic regression analysis, and health condition assessments to examine various determinants of CI.

**Results:**

The current study’s key findings demonstrate sub-statical correlations between CI and various factors, including educational attainment, marital status, and gender. Pronounced differences are evident between urban and rural demographics. Furthermore, aspects of social engagement, notably communication proficiency and sensory capabilities, exhibit a strong association with CI. Logistic regression analysis highlights that residing in rural areas (Odds Ratio [OR] = 0.637) and being female (OR = 0.71) are linked to a decreased risk of CI. In contrast, behavioral and health-related variables present a complex picture. Specifically, aggressive behavior (Adjusted OR = 1.881) and symptoms of depression (Adjusted OR = 0.549) contrast with conditions such as asthma (OR= 2.857) and cerebral infarction (OR=1.348), which elevate the risk of CI. Intriguingly, hyperlipidemia (OR= 0.671) appears to confer a protective effect against CI.

**Conclusion:**

The study highlights the complexity of factors affecting CI in the elderly, advocating for a comprehensive approach to understanding and managing cognitive health.

## 1 Introduction

Cognitive Impairment (CI) manifests as a spectrum of cognitive deficits in the elderly, impacting essential domains such as orientation, memory, calculation, attention, language, executive function, reasoning, and visualization abilities (1). This condition is broadly classified into mild cognitive impairment (MCI) and dementia, based on severity (2). Dementia, particularly prevalent among the older population, has emerged as a significant public health concern due to its substantial socio-economic and healthcare implications. For instance, in 2010, the prevalence of dementia in the U.S. population aged over 70 was approximately 14.7% (3).

In China, the incidence rate of dementia for individuals aged 60 and above is reported to be 9.9 cases per 1000 person-years. Alzheimer’s disease and related dementia show an increasing trend with age, evidenced by an 8% prevalence in those over 65, rising sharply to about 43% in adults over 85. Projections indicate that the number of individuals in the ‘oldest old’ category (over 80 years) will triple by 2050 (4). The prevalence of MCI, a transitional state between the normal cognitive aging process and early dementia, exhibits significant variability in China, with estimates ranging from 9.7% to 23.3% (5).

CI in older adults, a condition characterized by memory deficits, learning challenges, and impaired concentration, significantly compromises their quality of life. This impairment not only elevates the risk of dementia and mortality but also stems from a multifactorial etiology, including vascular issues, neuronal degeneration, and strokes (6). Empirical evidence indicates that around 10% of individuals who suffer strokes had pre-existing dementia, a figure that increases in post-stroke scenarios, especially among recurrent stroke patients (7). Central to its progression are factors such as gender (8), advancing age (9), smoking habits (10), limited mental and physical engagement (11), and reduced social interactions, which cumulatively exacerbate cognitive decline. Moreover, cardiovascular metabolic risk factors (12), including diabetes, hypertension, metabolic syndrome, and other vascular disorders (13), are strongly correlated with an acceleration of cognitive decline. Additional factors contributing to this impairment range from atrial fibrillation and depression to obesity (14), traumatic brain injury, hearing loss, alcohol misuse (15), and exposure to air pollution (16). The spectrum of cognitive impairment in the elderly spans from mild, often undiagnosed deficits to more severe conditions like dementia.

The Mini-Cog, a concise and innovative tool for dementia screening, combines two simple cognitive tasks: recalling three words and drawing a clock. Created by Borson and colleagues in 2000 (17)., this approach improves upon the Clock Drawing Test (CDT) and the Mini-Mental State Examination (MMSE), as evidenced by multiple studies. With an average administration time of just 3 minutes, it is faster than the MMSE and is preferred by elderly patients due to its omission of potentially uncomfortable orientation questions. It has been validated as an effective tool for screening dementia and cognitive impairment in various settings, including preoperative clinic evaluations.

Understanding the trajectory of cognitive impairment over a lifetime is crucial for developing strategies to prevent its onset and slow its progression towards dementia. However, data regarding the association of cognitive impairment with demographic factors, social engagement, and disease prevalence, particularly in individuals aged 80 and above, remains sparse. Our study aims to bridge this gap by investigating whether cognitive impairment, as detected using the Mini-Cog test, correlates with demographic characteristics, social engagement levels, and disease prevalence in older adults. To this end, we have designed a cohort study targeting adults aged 80 years and above.

## 2 Materials and methods

### 2.1 Methods

**Figure 1:**
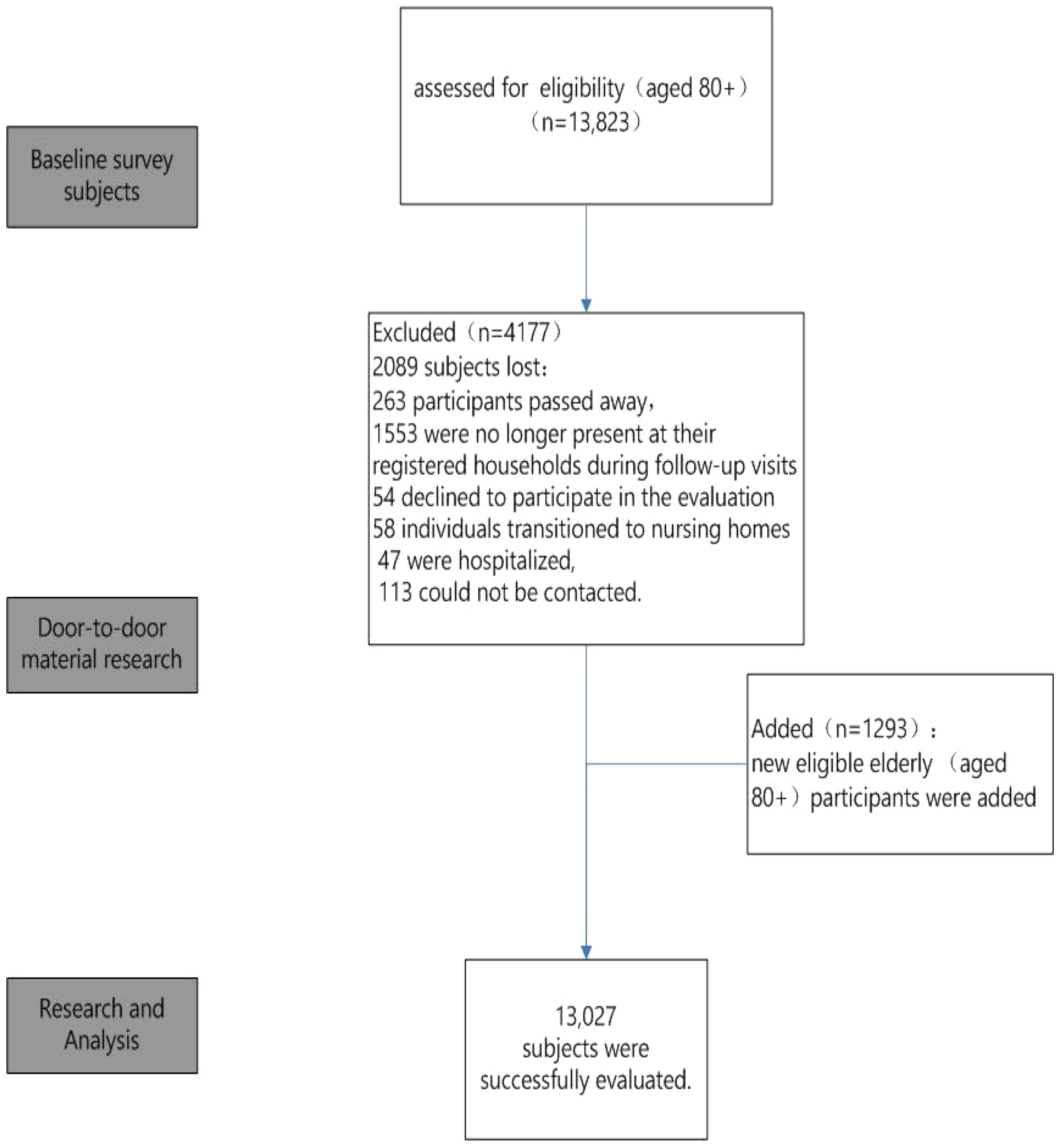
The Flowchart of the study.

Our research was a comprehensive 8-month-long (April to December 2022) population-based, cross-sectional study conducted in Lishui District, Jiangsu, China (**Figure 1**). The study commenced with an initial cohort of 13,823 individuals aged 80 or older, identified with the assistance of the Lishui Civil Affairs Bureau. The research team comprised a total of 57 researchers who underwent uniform training to ensure their proficiency in basic assessment knowledge and skills. This training was essential to guarantee that each of the 57 door-to-door researchers adhered to unified assessment requirements, thereby ensuring the scientific accuracy of the research data.

As the study progressed, an additional 1,293 elderly individuals were identified and incorporated, thereby expanding our study population. However, the dynamics of participant involvement underwent notable changes over the course of the study. Specifically, of the initial participant group, 263 individuals passed away, 1,553 were not found at their registered residences during follow-up visits, 54 opted to withdraw from the study, 58 relocated to nursing homes, 47 were hospitalized, and 113 remained unreachable for various reasons. After adjusting for these participant changes, the study successfully completed evaluations with a total of 13,027 elderly participants, as shown in **Figure 1**.

### 2.2 Measurements

The equations should be inserted in editable format from the equation editor.

#### 2.2.1 Cognitive impairment

In our study, we utilized the Mini-Cog assessment method as outlined by Borson et al. (15) to screen for cognitive impairment (18). This evaluation awards one point for each item correctly recalled and up to two points for the clock-drawing task, culminating in a maximum score of five points. Data were collected for both the recall and clock-drawing tasks for all participants. However, consistent with Borson’s scoring protocol, the clock-draw performance was scored only for patients who recalled between one and two words. ‘Cognitive Impairment’ was defined as a score of two or less, and ‘No Cognitive Impairment’ as a score greater than two, in accordance with the methodology established by Borson et al.

#### 2.2.2 Demographic factors and social engagement

This study rigorously explores the impact of various demographic and socioeconomic factors on the prevalence of Cognitive Impairment. These factors include age, occupation, urban-rural residency, marital status, educational level, income bracket, living conditions, and health insurance status. Concurrently, the study probes into the complex interplay of changes in social engagement and functional capabilities among the subjects. A comprehensive questionnaire was utilized, covering facets such as communication, sensory perception, life skills, occupational capacity, orientation in time and space, personal orientation, and social interaction.

#### 2.2.3 Disease survey questionnaire

To elucidate the correlation between CI and diverse medical conditions, a survey encompassing various diseases was executed. The surveyed conditions included hypertension, hyperglycemia, hyperlipidemia, cardiac disorders, cerebral infarction, leg and foot discomfort, pain, gastrointestinal diseases, back pain, stroke, pulmonary ailments, asthma, gout, osseous disorders, cancer, ocular conditions, renal diseases, gallstones, rheumatism, among others.

#### 2.2.4 Data analysis

In this investigation, we utilized SPSS software (IBM Corp., Armonk, NY, USA; Version 25.0.0.0) for a comprehensive analysis of the data. The threshold for statistical significance was established at p < 0.05. Depending on the nature of the measurement scales, group comparisons were executed using either Student’s t-tests for continuous variables or chi-square tests for categorical variables.

In our univariate analyses, we scrutinized variations in mini-cog scores across diverse demographic categories within the population aged 80 years and older. This analysis revealed significant disparities, highlighting the diversity within this age group. Moreover, the study assessed the predictive capability of mini-cog scores for the likelihood of disease onset using binary logistic regression analysis, applying a Forward Stepwise method. The significance level for this analysis was maintained at p < 0.05.

## 3 Results

### 3.1 Demographic factors in CI

These figures indicate the necessity for targeted approaches to address CI within various demographic segments (**Table 1**). The table details demographics and health characteristics of individuals with and without CI. In urban areas, 2244 individuals (75.6%) have no CI, while 6674 (66.4%) do. Rural areas have 725 (24.4%) without impairment and 3384 (33.6%) with it. Regarding marital status, the majority of non-impaired individuals are married (57.5%), while a significant proportion of impaired individuals are widowed (48.2%). For education, a notable 78.2% of cognitively impaired individuals are illiterate. In terms of income, most in both groups rely on wages/pension. Living situations show 34.5% of non-impaired and 34.7% of impaired individuals living alone. Gender-wise, more females (55.5%) have CI compared to males. Disease presence is nearly equal in both groups, and mental illness is found only among the cognitively impaired (4.1%).

**Table 1:** Demographic Factors in CI.

Variable Category	None CI(n(%)	CI (n(%)	Total (n(%)
Gender			
Male	1574 (53.0%)	4474(44.5%)	6048(46.4%)
Female	1395 (47.0%)	5584(55.5%)	6979(53.6%)
Urban/Rural			
Urban	2244 (75.6%)	6674(66.4%)	8918(68.5%)
Rural	725 (24.4%)	3384(33.6%)	4109(31.5%)
Marital Status			
Married	1682 (57.5%)	4803(50.1%)	6485(51.9%)
Widowed	1188 (40.6%)	4616(48.2%)	5804(46.4%)
Divorced	8 (0.3%)	15(0.2%)	23(0.2%)
Unmarried	26 (0.9%)	96(1.0%)	122(1.0%)
Unspecified	19 (0.7%)	50(0.5%)	69(0.6%)
Education			
Illiterate	1666 (60.2%)	7772(78.2%)	9438(74.3%)
Primary School	631 (22.8%)	1331(13.4%)	1962(15.4%)
Junior High	231 (8.3%)	426(4.3%)	657(5.2%)
High School/Tech/ Junior College	165 (6.0%)	309(3.1%)	474(3.7%)
College and Above	74 (2.7%)	98(1.0%)	172(1.4%)
Income Source			
Other Subsidies	206 (7.5%)	911(9.3%)	1117(8.9%)
Child Subsidies	409 (14.9%)	875(9.0%)	1284(10.2%)
Wages/Pension	2137 (77.7%)	7989(81.7%)	10126(80.8%)
Living Situation			
Living Alone	1024 (34.5%)	3487(34.7%)	4511(34.6%)
Living with Children	675 (22.7%)	2945(29.3%)	3620(27.8%)
Living with Spouse	1249 (42.1%)	3524(35.0%)	4773(36.6%)
Other	21 (0.7%)	102(1.0%)	123(0.9%)
Disease Presence			
No Disease	1660 (55.9%)	5553(55.2%)	7213(55.4%)
Disease	1309 (44.1%)	4504(44.8%)	5813(44.6%)

None CI (Cognitive Impairment Mini-Cog Score >2); CI (Cognitive Impairment Mini-Cog Score <=2);p < 0.01.

### 3.2 Association between social engagement aspects and CI

**Table 2** explored the association between various Social Engagement aspects and CI. We utilized a cross-tabulation approach, categorizing CI based on a scoring system. The findings revealed significant associations in several domains: Communication abilities (χ² = 780.122, p < 0.001), Vision (χ² = 1038.834, p < 0.001), Hearing (χ² = 1009.04, p < 0.001), Life Skills (χ² = 1095.233, p < 0.001), Work Ability (χ² = 1488.006, p < 0.001), Time/Space Orientation (χ² = 1552.55, p < 0.001), Person Orientation (χ² = 710.26, p < 0.001), and Social Interaction (χ² = 719.941, p < 0.001). No significant association was observed between the presence of disease and CI (χ² = 0.449, p = 0.5).

**Table 2:** Cross-Tabulation of Social Engagement and CI.

Category	None CI (n%)	CI (n(%)	Total	Chi-square (χ²)	p-value (p)
Presence of Disease					
none	16609 (55.9%)	5553 (55.2%)	7213	0.449	0.5
Have one or more	1309 (44.1%)	4504 (44.8%)	5813		
Communication					
No difficulty in communicating with others normally	2279 (76.8%)	4857 (48.3%)	7136	780.122	0.001
Some Difficulty; Able to express needs and understand others but requires more time or assistance	558 (18.8%)	3549 (35.3%)	4107		
Difficulty in expressing needs or understanding others, frequent repetition or simplified verbal expression needed.	113 (3.8%)	1236 (12.3%)	1349		
Unable to express needs or understand others	19 (0.6%)	416 (4.1%)	435		
Vision					
Can Read Standard Text; Able to read standard text in books and newspapers clearly.	875 (29.5%)	904 (9%)	1779	1038.834	0.001
Can Read Large Text but unable to read standard text in books and newspapers clearly	1372 (42.6%)	4339 (43.1%)	5711		
Limited Vision, unable to read newspaper headlines clearly but can recognize objects	647 (21.8%)	3790 (37.7%)	4437		
Difficulty Recognizing Objects, but eyes can follow object movement, only able to see light, colors, and shapes	65 (2.2%)	854 (8.5%)	919		
No Vision, eyes cannot follow object movement.	10 (0.3%)	171 (1.7%)	181		
Hearing					
Normal Conversation	1716 (57.8%)	2783 (27.7%)	4499	1009.04	0.001
Inaudible in Quiet: Can have a normal conversation and hear sounds from the TV, phone, and doorbell	857 (28.9%)	3811 (37.9%)	4668		
Difficulty in Normal Settings: Unable to hear clearly when speaking softly or when the speaker is more than 2 meters away.	280 (9.4%)	2058 (20.5%)	2338		
Partial Hearing with Loud Sound, Some difficulty in normal communication, needs a quiet environment or loud speech to hear.	100 (3.4%)	1221 (12.1%)	1321		
Partial Hearing with Slow Sound: Can hear partially only when the speaker speaks loudly or slowly.	16 (0.5%)	185 (1.8%)	201		
Life Skills					
Beyond Personal Care: Can manage household chores and home affairs in addition to personal self-care (e.g., eating, grooming, dressing).	1634 (55%)	2473 (24.6%)	4107	1095.233	0.001
Household Chores, but Poorer: Can do household chores but not well-organized, lacks order in household affairs.	899 (30.3%)	3972 (39.5%)	4871		
Basic Self-Care, Poor Quality: Personal basic self-care (e.g., eating, toileting) is possible, but household chores can only be done with the help of others, and quality is poor.	303 (10.2%)	1770 (17.6%)	2073		
Basic Self-Care, Assistance: Able to manage personal basic life affairs (e.g., eating, toileting) and can wash up with reminders.	50 (1.7%)	813 (8.1%)	863		
Dependence on Others: Partial help needed for personal basic life affairs (e.g., eating, toileting) or total reliance on others.	83 (2.8%)	1030 (10.2%)	1113		
Work Ability					
Skilled Work as Usual: Formerly skilled mental or physical work can be performed as usual.	1063 (35.8%)	971 (9.7%)	2034	1488.006	0.001
Reduced Work Ability: Reduced ability in formerly skilled mental or physical work.	1284 (43.2%)	4156 (41.3%)	5440		
Significant Work Decline: Formerly skilled mental or physical work is significantly worse than before, with some forgetting.	483 (16.3%)	2856 (28.4%)	3339		
Fragmented Skills: Only some fragments of previously skilled work are retained, and all skills are forgotten.	100 (3.4%)	1064 (10.6%)	1164		
Total Skill Erosion: All previous knowledge or skills have been erased.	39 (1.3%)	1011 (10.1%)	1050		
Time/Space Orientation					
Clear Concept: Clear concept of time (year, month, day, hour) and can go out alone, quickly grasping the orientation of new environments.	2202 (74.2%)	3399 (33.8%)	5601	1552.55	0.001
Near Streets, No Route Home: Concept of time has declined slightly, can go out alone in nearby areas but does not know the way back home.	538 (18.1%)	3903 (38.8%)	4441		
Poor Time Concept	141 (4.7%)	1294 (12.9%)	1435		
Very Poor Time Concept	39 (1.3%)	524 (5.2%)	563		
No Time Concept	49 (1.7%)	938 (9.3%)	987		
Person Orientation					
Recognizes People and Age: Knows the relationships of people around and can roughly estimate the age and identity of strangers.	2599 (87.5%)	6221 (61.9%)	8820	710.26	0.001
Knows Intimate Family Only: Only knows the relationships of close family members, unable to estimate the age of strangers, cannot address strangers.	277 (9.3%)	2412 (24%)	2689		
Calls Family Members, No Relation: Can only address family members, or can only address people the same way without knowing their relationships or generations.	48 (1.6%)	415 (4.1%)	463		
Recognizes Some Relatives: Only recognizes commonly living relatives, can address children or grandchildren, can distinguish between familiar and unfamiliar people.	37 (1.2%)	604 (6%)	641		
Recognizes Guardians Only: Only recognizes caregivers, cannot distinguish between familiar and unfamiliar people.	8 (0.3%)	406 (4%)	414		
Social Interaction					
Active Participation: Participates in society, has a certain adaptability in the social environment, and interacts appropriately with people.	2192 (73.8%)	4670 (46.4%)	6862	719.941	0.001
Participates Passively, Vulnerable: Disconnected from society, can have passive interactions but does not initiate interactions, uses inappropriate words and phrases in conversations, easily deceived.	538 (18.1%)	3197 (31.8%)	3735		
Engages with Difficulty: Can barely interact with people, unclear speech content, inappropriate expressions.	151 (5.1%)	1137 (11.3%)	1288		
Difficulty in Interaction: Difficulty in interacting with people.	69 (2.3%)	593 (5.9%)	662		
Difficulty in Socialization: Difficulty in socialization.	19 (0.6%)	461 (4.6%)	480		

None CI(Cognitive Impairment Mini-Cog Score >2);CI (Cognitive Impairment Mini-Cog Score <=2);p < 0.01.

### 3.3 Demographic, economic, social CI influences

In our logistic regression analysis examining the impact of various factors on CI (**Table 3**), significant associations were found across demographic, behavioral, health, insurance, work ability, and social relationship variables. Residence in a rural area was associated with a decreased likelihood of the outcome, as evidenced in both unadjusted (OR = 0.637, 95% CI (0.58, 0.7) and adjusted models (OR = 0.672, 95% CI (0.595, 0.759), with statistical significance (p < 0.001). Similarly, female gender was significantly linked to a lower likelihood of the outcome in both models (Unadjusted: OR = 0.71, 95% CI (0.654, 0.771); Adjusted: OR = 0.708, 95% CI (0.652, 0.769), p < 0.001). Income sources showed varying impacts; income for children was significant only in the unadjusted model (OR = 1.183, 95% CI (1.009, 1.386), p = 0.038), while pension income showed a strong negative association in both models.

**Table 3:** Demographic, Economic, Social Factors associated with CI.

Variable	Model a Exp(B) & 95% CI	p	Model b Exp(B) & 95% CI	p
Residence (Urban/Rural)				
Rural	0.637 (0.58, 0.7)	0.001	0.672 (0.595, 0.759)	0.001
Gender				
Female	0.71 (0.654, 0.771)	0.001	0.708 (0.652, 0.769)	0.001
Income (None or Government Aid)				
Income from Children	1.183 (1.009, 1.386)	0.038	1.077 (0.89, 1.303)	0.448
Income with Pension	0.572 (0.504, 0.65)	0.001	0.361 (0.306, 0.427)	0.001
Aggressive Behavior				
Has Aggressive Behavior (1)	0.477 (0.31, 0.733)	0.001	1.881 (1.082, 3.27)	0.025
Depression Symptoms (1)	0.192 (0.125, 0.296)	0.001	0.549 (0.32, 0.944)	0.03
Basic Health Condition				
Has Disease	0.972 (0.895, 1.056)	0.503	1.304 (1.172, 1.45)	0.001
Social Insurance - Urban Medical				
New Rural Cooperative Medical	0.328 (0.269, 0.399)	0.001	0.316 (0.243, 0.41)	0.001
Urban Resident Medical Insurance	0.565 (0.477, 0.669)	0.001	0.442 (0.362, 0.54)	0.001
Work Ability - Normal Activities				
Some Decline	0.035 (0.025, 0.049)	0.001	0.214 (0.139, 0.329)	0.001
Markedly Worse, Partial Forget	0.125 (0.09, 0.173)	0.001	0.289 (0.191, 0.435)	0.001
Partial Retention, Skills Lost	0.228 (0.163, 0.319)	0.001	0.458 (0.305, 0.687)	0.001
Completely Forget	0.41 (0.281, 0.6)	0.001	0.722 (0.469, 1.111)	0.139
Social Relationships - Age and Identity				
Cannot Discern Age, Cannot Address Strangers	0.047 (0.023, 0.095)	0.001	0.379 (0.163, 0.883)	0.025
Can Only Address Family Members or Use Familiar Terms, Do Not Know Their Relationship or Generation	0.172 (0.084, 0.349)	0.001	0.351 (0.151, 0.815)	0.015
Only Recognize Household Members, Can Address Children or Grandchildren, Can Distinguish Familiar and Strangers	0.17 (0.08, 0.365)	0.001	0.166 (0.069, 0.399)	0.001
Only Recognize Guardians, Cannot Distinguish Familiar and Strangers	0.322 (0.148, 0.698)	0.004	0.317 (0.134, 0.751)	0.009

Model A: unadjusted; Model B is an adjusted model, incorporating all relevant variables(Residence, Gender, Income, Aggressive Behavior, Basic Health Condition, Social Insurance,Work Ability, Social Relationships); CI: confidence interval; (p ≤ 0.05).

Behavioral and health factors presented contrasting associations with the outcome. Aggressive behavior increased the likelihood of the outcome (Adjusted OR = 1.881, 95% CI (1.082, 3.27), p = 0.025), whereas depression symptoms were inversely associated (Adjusted OR = 0.549, 95% CI (0.32, 0.944), p = 0.03). Basic health conditions, specifically having a disease, were not significant in the unadjusted model but showed a significant positive association after adjustment (OR = 1.304, 95% CI (1.172, 1.45), p < 0.001). Participants with New Rural Cooperative Medical Insurance or Urban Resident Medical Insurance were notably less likely to experience the outcome, with the New Rural Cooperative Medical group showing the most substantial effect (Adjusted OR = 0.316, 95% CI (0.243, 0.41), p < 0.001).

Work ability and social relationships also demonstrated significant impacts. A marked decline in work ability and deterioration in social relationships were strongly associated with the outcome. The most pronounced effects were observed in participants with markedly worse work ability (Adjusted OR = 0.214, 95% CI (0.139, 0.329), p < 0.001) and those only able to recognize household members (Adjusted OR = 0.166, 95% CI (0.069, 0.399), p < 0.001). These findings emphasize the multifaceted nature of CI and the importance of considering a broad range of factors in its understanding and management.

### 3.4 Disease factors influencing CI

Our analysis identified significant associations between multiple health conditions and CI, as presented in **Table 4**. Asthma and Cerebral Infarction showed a strong positive association with the CI. In both unadjusted and adjusted models, individuals with asthma had approximately 2.86 times higher odds of experiencing CI (OR= 2.857, 95% CI(1.435, 5.686); OR =2.863, 95% CI(1.436, 5.709). Similarly, cerebral infarction was linked to an increased likelihood of CI (Unadjusted OR=1.348, 95% CI(1.118, 1.625); Adjusted OR=1.368, 95% CI(1.13,1.657).

**Table 4:** Disease Factors Influencing CI.

Health Conditions	Model A Exp(B) & 95% CI	P	Model B Exp(B) & 95% CI	P
Difficulty in Leg Movement	1.42(1.101, 1.83)	0.007	1.408(1.091, 1.817)	0.009
Asthma	2.857(1.435, 5.686)	0.003	2.863(1.436, 5.709)	0.003
Cerebral Infarction	1.348(1.118, 1.625)	0.002	1.368(1.13, 1.657)	0.001
Hyperlipidemia	0.705(0.484, 1.028)	0.069	0.671(0.458, 0.985)	0.042
Waist/Lower Back Condition	0.578(0.4, 0.834)	0.003	0.581(0.402, 0.839)	0.004

Model A: unadjusted; Model B: adjusted for Difficulty in Leg Movement,Asthma,Cerebral Infarction,Hyperlipidemia,Waist/Lower Back Condition CI: confidence interval; (p ≤ 0.05).

Conversely, Hyperlipidemia and Waist/Lower Back Condition were associated with a reduced likelihood of CI. Hyperlipidemia showed a significant protective effect in the adjusted model (OR= 0.671, 95% CI(0.458,0.985), though it was not significant in the unadjusted model. The waist/lower back condition consistently showed a negative association in both models (Unadjusted OR=0.578, 95% CI(0.4, 0.834); Adjusted OR=0.581, 95% CI(0.402,0.839). Difficulty in Leg Movement was also found to moderately increase the likelihood of CI (Unadjusted OR=1.42, 95% CI(1.101,1.83); Adjusted OR=1.408, 95% CI(1.091,1.817). These findings highlight the varied influence of different health conditions on the risk of CI, underscoring the need for tailored approaches in its management and prevention.

## 4 Discussion

These figures highlight key differences and potential correlations between gender and various such as social (19), educational, and health factors with CI (20), where social factors (21) include urban-rural residence, marital status, income source, living conditions. The urban-rural divide (22) in cognitive scores could be attributed to differences in access to healthcare, educational opportunities, and lifestyle factors. Marital status, particularly being married, seems to confer a protective effect on cognitive health, potentially due to social support and shared responsibilities (23).

The strong link between educational attainment (24) and cognitive function underscores the importance of education in cognitive health. Higher education levels may provide better cognitive stimulation and access to information on health-promoting behaviors. Income source, particularly from pensions or wages, suggests a possible link between financial stability and cognitive health (25).

Living arrangements’ influence on cognitive function highlights the role of social interactions and support systems in maintaining cognitive health (26). The gender differences observed point towards potential biological and social factors influencing cognitive health.

There is a complex relationship between health condition and functional abilities among the study participants. While certain domains, such as communication, vision, hearing, life skills, work ability, time/space orientation, person orientation, and social interaction, showed significant associations with functional abilities, the presence of disease did not exhibit a significant impact. These findings highlight the multifaceted nature of functional abilities and the need for a comprehensive assessment.

CI or dementia refers to a clinical syndrome characterized by challenges in memory, language, and behavior, resulting in limitations in performing daily activities. Moreover, the results of this study reveal that the capacity to execute daily life skills (27), maintain occupational effectiveness, and preserve orientation in time and space emerged as notable determinants of functional abilities.

The significant association of rural residence with a decreased likelihood of the outcome contrasts with common perceptions and may reflect underlying socioeconomic and healthcare disparities (28). Gender differences, particularly the lower likelihood of the outcome among females, align with previous studies suggesting gender-specific risk factors and resilience mechanisms. The differential impact of income sources, notably the significant negative association of pension income, could be indicative of socioeconomic stability’s influence on health outcomes.

The contrasting associations of behavioral factors, such as aggressive behavior (29) and depression (30) symptoms, with the outcome underscore the complexity of behavioral health’s role. The significant association after adjustment for having a disease highlights the importance of considering comorbid conditions in evaluating health outcomes.

The substantial impact of social insurance types, especially the New Rural Cooperative Medical Insurance, suggests that healthcare accessibility and quality are critical determinants of health outcomes. These findings emphasize the importance of functional and social aspects in health, going beyond mere clinical or behavioral considerations. This aligns with the growing recognition of holistic health approaches, integrating physical, mental, and social well-being.

The results of this study underscore that respiratory and circulatory conditions (31), such as asthma (32) and cerebral infarction (33), significantly elevate the risk of CI. The observed increase in odds ratios for asthma and cerebral infarction emphasizes the need for vigilant monitoring and management in patients with these conditions.

Conversely, our study revealed a notable protective effect of hyperlipidemia and waist/lower back conditions (34) against CI. This counterintuitive finding, particularly for hyperlipidemia, which is often considered a risk factor for various health issues (35), suggests a potential area for further investigation. It raises questions about the underlying mechanisms that might confer this protection and how these might be leveraged in clinical practice.

The moderate increase in the likelihood of CI associated with difficulty in leg movement (36) could be indicative of broader mobility or lifestyle-related factors impacting health outcomes. This observation aligns with studies emphasizing the importance of physical mobility in overall health (37).

This study conducted an extensive door-to-door survey, meticulously targeting individuals aged over 80 years within a specific locale, with the objective of performing a thorough evaluation. The investigation revealed a range of demographic, social, and health challenges prevalent among older adults living in their own homes and examined their association with CI. This research thus offers a foundational dataset for future studies. It is crucial to acknowledge the limitations of this study, notably its focused demographic and methodological approach. Further investigation into the health status disparities between elderly individuals residing independently and those in nursing homes or healthcare facilities is essential. Additionally, the selection of participants should incorporate considerations of regional differences.

## 5 Conclusion

The study reveals correlation between CI and demographic and health factors. Urban-rural differences are evident, for social engagement playing a significant role. Logistic regression analysis highlights rural residence and female gender as protective against CI, while behavioral and health factors present a complex picture. Hyperlipidemia shows a protective effect. These findings underscore the multifaceted nature of CI and the importance of comprehensive assessment and intervention strategies.

## Data availability statement

The original contributions presented in the study are included in the article/supplementary material. Further inquiries can be directed to the corresponding author.

## Ethics statement

Approval for this study was granted by the Ethical Committee of Jiangsu SuYi Health Care Research Institute (1/4/2022). The studies were conducted in accordance with the local legislation and institutional requirements. The participants provided their written informed consent to participate in this study.

## Author contributions

SB: Writing – review & editing, Writing – original draft, Resources, Methodology, Investigation, Formal Analysis, Data curation, Conceptualization. XT: Writing – review & editing, Supervision, Project administration, Investigation, Funding acquisition, Conceptualization. FM: Writing – review & editing, Methodology, Conceptualization. CX: Writing – review & editing, Project administration, Investigation, Data curation. YZ: Writing – review & editing, Investigation. QG: Writing – review & editing, Investigation. CB: Writing – review & editing, Investigation.
